# Editorial: Patient cooperative robotics in neurorehabilitation

**DOI:** 10.3389/fnbot.2023.1211287

**Published:** 2023-05-17

**Authors:** Imre Cikajlo, Fabrizio Patane, Sehoon Oh

**Affiliations:** ^1^Research and Development Unit, University Rehabilitation Institute Soča, Ljubljana, Slovenia; ^2^Faculty of Engineering, University Niccoló Cusano, Rome, Italy; ^3^Department of Robotics Engineering, Daegu Gyeonbuk Institute of Science and Technology, Daegu, Republic of Korea

**Keywords:** neurorehabilitation, robotics, electromyography, exoskeleton, gait, upper extremities

In recent years, there has been a growing interest in the use of robotics in neurorehabilitation, particularly in the treatment of patients with neurological disorders. One of the most promising applications of robotics in this field is patient cooperative robotics, which involves the use of robotic devices that work in collaboration with patients to assist them in their rehabilitation. By working in collaboration with patients, these devices can help them to regain movement and function, as well as to improve their overall quality of life. Wang et al. presented a gait exercise assist robot (GEAR), intended for post-stroke patients to improve their gait. The authors reported on promising results, but emphasized the importance of further long-term clinical studies. One of the key advantages of patient cooperative robotics is that it allows for a more personalized approach to neurorehabilitation. Because the devices are designed to work in collaboration with patients, they can be tailored to the specific needs of each individual, taking into account their unique abilities and limitations. Robotics exoskeletons (Nolan et al.) are designed as bilateral actuators for knee and hip joints and can provide support according to the patient's individual requirements. The authors' study in acute post-stroke individuals demonstrated the differences in functional recovery between the group receiving conventional standard treatment and the group receiving also additional robotic exoskeleton training. Another advantage of robotic supported treatment is that it can help to reduce the burden on healthcare professionals. By automating certain aspects of the rehabilitation process, these devices can free up time and resources for healthcare professionals, allowing them to focus on other important aspects of patient care. Karunakaran et al. contributed a review of applications of robotic exoskeletons in acquired brain injury. This review identifies the gaps in research and suggest some recommendations. Despite its many advantages, patient cooperative robotics is still a relatively new field, and there is much work to be done in order to fully realize its potential. In particular, more research is needed to better understand how these devices can be used to improve the outcomes of neurorehabilitation, and to identify the most effective ways to integrate them into existing rehabilitation programs. E.g. Nakagawa et al. presented an active robotic exoskeleton for ankle joint that identifies the phases of gait, controls the angular velocity and generates the required ankle joint torque. The authors's study also assessed electromyography (EMG) in dorsiflexors and plantarflexors, but included neuromuscularly intact volunteers. The technology continues to evolve and improve, it has the potential to revolutionize the way that we approach the treatment of patients with neurological disorders, and to help them to achieve better outcomes and a higher quality of life.

In addition to patient cooperative robotics, another promising area of research in neurorehabilitation is human-robot interaction using EEG (electroencephalography) and EMG signals to detect and interpret the patient's intentions for movement (Zhang et al.). EEG measures the electrical activity in the brain and can be used to detect changes in brain activity related to movement intention. EMG measures the electrical activity of muscles and can be used to detect muscle activation related to movement intention. The authors combined these signals and developed a system that can interpret the patient's lower limb voluntary intention for movement. Numerical simulations were provided with limited number of participants. The outcomes may present a valuable information that can be translated into robotic movements, allowing for more natural and intuitive interaction between the patient and the robot. Saga et al. provided a clarification of EEG features coming directly from the brain computer interface (BCI) during ankle dorsiflexion using the pneumatic ankle rehabilitation system. The outcomes with six neurologically intact and a single participant with hemiplegia demonstrated the activity of the primary motor area and somatosensory area of the brain.

Such technology has the potential to greatly enhance the effectiveness of neurorehabilitation by providing more targeted and personalized therapy, as well as allowing for more frequent and intensive training. It can also help to reduce the burden on healthcare professionals by automating certain aspects of the rehabilitation process. However, there are still many challenges to be addressed in the development of these systems. One of the biggest challenges is the variability in EEG and EMG signals between patients, which can make it difficult to develop a system that is accurate and reliable for all patients. Additionally, the complexity of interpreting these signals requires advanced machine learning algorithms and sophisticated signal processing techniques. Despite these challenges, the potential benefits of human-robot interaction using EEG and EMG signals are significant, and ongoing research in this area holds great promise for the future of neurorehabilitation.

## Author contributions

IC edited and wrote the editorial. FP edited and contributed to the text. SO reviewed and complemented the editorial. All authors contributed to the article and approved the submitted version.

